# Trigeminocardiac reflex in bimaxillary orthognathic surgery: case review

**DOI:** 10.4317/medoral.27062

**Published:** 2025-04-06

**Authors:** Luis Ortiz-Peces, María Álvaro-Martínez, Álvaro Damián Moreiras-Sánchez, Guillermo Chacón-Ferrer, Martín Andura-Correas, José Luis del Castillo-Pardo de Vera, Luis Ortiz-González, José Luis Cebrián-Carretero

**Affiliations:** 1 Department of Oral and Maxillofacial Surgery, La Paz University Hospital. Madrid, Spain; 2 Department of Biomedical Sciences, Faculty of Medicine and Health Sciences, University of Extremadura. Badajoz, Spain

## Abstract

**Background:**

The trigeminocardiac reflex (TCR) is a rare but clinically significant phenomenon characterized by bradycardia, hypotension, or asystole triggered by trigeminal nerve stimulation during maxillofacial surgery. It necessitates prompt recognition and management to ensure patient safety. TCR has been reported in orthognathic surgery, particularly during specific surgical maneuvers.

**Material and Methods:**

We report the case of a 36-year-old male who experienced TCR during bimaxillary orthognathic surgery. Detailed documentation of the patient’s clinical characteristics, intraoperative events, and management strategies was included. Additionally, we conducted a systematic review of the literature using Medline, Embase, Web of Science, and Scopus databases to identify cases of TCR in orthognathic surgery published from 1989 onward. Keywords included "trigeminocardiac reflex," "orthognathic surgery," "Le Fort I," and "bilateral sagittal split osteotomy".

**Results:**

We present the case of a patient who experienced transient bradycardia and asystole during mandibular manipulation and pterygomaxillary disjunction. The episode was successfully managed with atropine and cessation of triggering maneuvers. Additionally, a systematic review identified 10 cases of TCR in orthognathic surgery, most of which occurred during Le Fort I osteotomies, particularly during maxillary downfracture, followed by bilateral sagittal split osteotomies. Common manifestations included bradycardia and asystole. Management strategies involved cessation of surgical stimuli, administration of anticholinergic agents, and, in one severe case, cardiopulmonary resuscitation.

**Conclusions:**

TCR in orthognathic surgery is a significant risk requiring vigilance and prompt management. Understanding its triggers, maintaining intraoperative monitoring, and employing preventive strategies, such as gentle manipulation and proper anesthesia protocols, are essential for optimizing patient safety.

** Key words:**Trigeminocardiac reflex, orthognathic surgery, Le Fort I osteotomy, bilateral sagittal split osteotomy, asystole, bradycardia.

## Introduction

The trigeminocardiac reflex (TCR) is a significant physiological response characterized by bradycardia, hypotension, or even asystole, triggered by stimulation of the trigeminal nerve branches during surgical procedures in the maxillofacial region. This reflex, also known as the trigeminovagal reflex, has been described as a mechanism resulting from the interaction between the trigeminal nervous system and the autonomic cardiac reflex. Activation of the TCR involves transmission through the trigeminal spinal nucleus, which connects with the dorsal vagal nucleus and triggers a vagal response that reduces heart rate and blood pressure (1,2).

Historically, the TCR was initially identified as a risk event in ophthalmic surgery under the name oculocardiac reflex. However, studies such as those by Bohluli *et al*. have expanded knowledge of this reflex in maxillofacial surgery, showing its occurrence in interventions involving direct manipulation of cranial and facial structures, such as osteotomies and reconstructive procedures (1). This phenomenon has been documented in facial trauma surgeries, zygomatic arch fracture repairs, temporomandibular joint (TMJ) procedures, as well as in Le Fort I osteotomies and bilateral sagittal split osteotomies (BSSO).

The incidence of TCR in orthognathic surgery underscores the importance of closely monitoring the patient’s vital signs during critical moments of the procedure, with a preventive approach focusing on controlled surgical techniques and appropriate anesthesia. Following an incident at our institution, this article aims to analyze the cases reported in the literature and provide a comprehensive perspective on the intraoperative moments in orthognathic surgery when TCR most frequently occurs, with the goal of optimizing patient safety through effective collaboration between the surgical and anesthesia teams.

## Material and Methods

This study includes two components: the presentation of a clinical case of trigeminocardiac reflex (TCR) observed at our institution and a systematic review of the literature.

We first report the case of a 36-year-old male who experienced TCR during bimaxillary orthognathic surgery performed at our service. Detailed documentation of the patient’s clinical characteristics, intraoperative events, and management strategies was included for analysis and discussion within the context of previously reported cases.

Subsequently, a systematic literature search was conducted to identify eligible articles related to the trigeminocardiac reflex (TCR) in the context of orthognathic surgery. The search included the databases Medline, Embase, Web of Science (WOS), and Scopus, covering cases published from 1989 onward. The terms "trigeminocardiac reflex" were combined with keywords related to orthognathic surgery: "Orthognathic Surgery," "Le Fort I," and "Bilateral Sagittal Split Osteotomy." Only cases of TCR reported in orthognathic surgeries were included, excluding those associated with neurosurgical, otorhinolaryngological procedures, or any maxillofacial surgery interventions other than orthognathic surgery.

## Results

A 36-year-old Caucasian male was scheduled for bimaxillary orthognathic surgery to address Class III malocclusion and facial asymmetry. He had no history of toxic habits or regular medications, with grade 1 obesity as his only relevant medical history. Preoperative evaluation showed no significant findings, and his preanesthetic assessment classified him as ASA II.

The surgery followed standard protocol, involving bilateral mandibular ramus osteotomy and Le Fort I osteotomy. Total intravenous anesthesia (TIVA) was administered, with induction using 5 mg midazolam, 300 mcg fentanyl, 80 mg lidocaine, 1% propofol in target-controlled infusion, and an initial dose of 50 mg rocuronium plus an additional 40 mg at the procedure's start. Maintenance was achieved with 60% oxygen in air, 1% propofol, and dexmedetomidine at 2 mcg/mL infused at 4-8 mL/h. Nasotracheal intubation was performed using direct videolaryngoscopy. Fifteen minutes prior to starting, 10 cc of 1% lidocaine with 1:100,000 adrenaline was infiltrated bilaterally into the mandibular branches to block the inferior alveolar, lingual, and buccal nerves. Additionally, local anesthesia was infiltrated into the upper vestibular fold fifteen minutes before starting the Le Fort I osteotomy.

During the procedure, the patient maintained sinus rhythm, with an average heart rate of 65 beats per minute and blood pressure of 100/65 mm Hg. Upon completing the right mandibular ramus osteotomy and identifying the inferior alveolar nerve, a full disjunction maneuver was performed. At this moment, the patient experienced a significant drop in heart rate to 35 beats per minute, which recovered rapidly upon stopping the maneuver. The same response occurred on the contralateral side, with the heart rate recovering adequately after maneuver interruption.

In the maxilla, during the downfracture, a pterygomaxillary disjunction assisted with a Turvey-type disjunctor was performed, at which point the patient experienced a sharp drop in heart rate, leading to asystole. Immediately halting the maneuver resulted in the initiation of an idioventricular rhythm within 5 seconds, which subsequently transitioned to sinus rhythm. A dose of 0.5 mg atropine was administered, increasing the heart rate to 95 beats per minute, without noTable bleeding or any further sudden drops in heart rate for the remainder of the procedure. The surgery was successfully completed.

In the immediate postoperative period, the patient experienced a single episode of vomiting, and the rest of his recovery was uneventful. He reported no symptoms compatible with a vasovagal reflex during the two-month follow-up.

In our review, a total of 10 patients with manifestations of the trigeminocardiac reflex (TCR) during orthognathic surgery were identified (Table 1). Of these, four cases occurred in monomaxillary surgeries, while six were recorded in bimaxillary surgeries. Clinical manifestations of TCR included seven episodes of asystole and four of bradycardia (3-9).

In terms of osteotomy types, seven cases occurred during Le Fort I osteotomies, while three were associated with bilateral sagittal split osteotomies (BSSO). Additionally, one instance of asystole was observed during the initial use of a mouth prop in the procedure's early phase (7). Notably, no publications on TCR in orthognathic surgery were found between 1994 and 2019, suggesting a possible underreporting or lack of awareness of this phenomenon during that period.

In all cases, surgical manipulation was immediately halted upon identification of TCR, allowing for recovery of sinus rhythm in all patients. Atropine was administered in six cases, glycopyrrolate in three cases, and in one specific instance, lidocaine was used in combination with glycopyrrolate (3-9). In one prolonged episode of asystole, the medical team initiated cardiopulmonary resuscitation (CPR) (8).

These results highlight the importance of vigilant monitoring and prompt management of TCR, demonstrating that cessation of manipulation and the use of anticholinergic agents such as atropine and glycopyrrolate are effective in resolving this reflex in the surgical context.

## Discussion

The trigeminocardiac reflex (TCR) is a physiopathological phenomenon triggered by stimulation of the trigeminal nerve branches, generating a vagal response that can lead to bradycardia, asystole, and other significant hemodynamic changes. This response follows an afferent pathway connecting the trigeminal nucleus to the motor nucleus of the vagus nerve, resulting in a reduction in heart rate and blood pressure (2). This connection between the trigeminal and cardiac systems, as highlighted by Chowdhury *et al*., is particularly relevant in the context of cranio-maxillofacial procedures where manipulation of trigeminal nerve branches occurs (1,2).

In our review, a higher prevalence of asystole than bradycardia was found, particularly during Le Fort I osteotomies compared to BSSO procedures (Table 1) (3-9). These findings align with previous studies, such as that by Bohluli *et al*., who reported that pterygomaxillary disjunction and maxillary downfracture during Le Fort I osteotomy are critical moments for TCR activation due to the stimulation of the palatine or alveolar branches of the trigeminal nerve (10). This type of manipulation appears to directly trigger the reflex, as the maxillary branches of the trigeminal nerve are in close contact with the surgical area. Kiani *et al*. also observed noTable hemodynamic changes during maxillary downfracture in Le Fort I, supporting the hypothesis that maxillary nerve manipulation is a key trigger for TCR in these procedures (11).

The particularity of the case we have reported underscores that the occurrence of hemodynamic changes compatible with vagal reflexes during certain movements in orthognathic surgery should alert the surgical team to the possibility of asystole during the procedure. Early identification and vigilance are essential to mitigate this risk in bimaxillary orthognatic surgery.

An important aspect of this review is the identification of potential biases and limitations in the available literature. There is a reporting bias favoring more severe cases, such as episodes of asystole, while milder or transient cases of bradycardia may go unrecorded. Lubbers *et al*. indicate that this bias is common in maxillofacial literature, where less severe events are less likely to be published (12). Additionally, the absence of studies on TCR in orthognathic surgery between 1994 and 2019 suggests that TCR might be underreported, limiting a comprehensive understanding of its prevalence and manifestations during orthognathic surgery (13).

Regarding the management of TCR, our review shows that the immediate cessation of surgical manipulation is an effective measure to allow sinus rhythm recovery. In several reported cases, administration of anticholinergics, such as atropine and glycopyrrolate, has been used to reverse TCR effects, especially in episodes of asystole or severe bradycardia (3-7,9). Additionally, Bohluli *et al*. (2011) suggest that mandibular nerve block using the Gow-Gates technique may reduce the incidence of TCR in procedures like BSSO, as this block minimizes the peripheral response of the reflex (14). However, while this approach appears promising, it does not completely eliminate the risk of TCR activation, indicating that trigeminal nerve branch manipulation remains a critical factor.

From a preventive perspective, some authors have recommended specific strategies. Besides peripheral nerve blocks, it is advisable to avoid excessive pressure in critical areas such as the pterygomaxillary region during Le Fort I downfracture (10). Constant monitoring of vital signs, with special attention to heart rate, is essential to rapidly detect the onset of TCR in these types of procedures. As demonstrated in our reported case, a sudden drop in heart rate during surgery should prompt an alert for possible asystole. Upon any unusual hemodynamic change, immediate suspension of manipulation is the first recommended response to minimize the risk of severe complications.

In orthognathic surgery, close coordination between the surgical and anesthesia teams is essential to anticipate the possibility of TCR. Additionally, the use of local anesthesia techniques, gentle and controlled manipulation, and precise instrumentation can be effective measures to reduce the likelihood of triggering this reflex.

## Figures and Tables

**Table 1:** Patient characteristics, surgical timing, and management of reported TCR cases in the English literature.

First Author *et al*. (Year)	Gender, Age, Race	Type of Surgery	Moment of TCR	Manifestation	Resolution
Ragno *et al*. (1989) (3)	Male, 17, Caucasian	Monomaxillary (Le Fort I)	During downfracture of the maxilla	Asystole	Stopped surgery, atropine, glycopyrrolate
Lang *et al*. (1991) - Case 1 (4)	Female, 28, Caucasian	Monomaxillary (Le Fort I)	Pressure with osteotome in pterygomaxillary osteotomy	Asystole	Atropine, release pressure
Lang *et al*. (1991) - Case 2 (4)	Female, 26, Caucasian	Bimaxillary	Placement of retractor during sagittal split mandibular osteotomy	Asystole	Stopped procedure, atropine, nerve block
Lang *et al*. (1991) - Case 3 (4)	Female, 38, Caucasian	Monomaxillary (Le Fort I)	Forward traction of the maxilla	Bradycardia	Stopped traction
Campbell *et al*. (1994)​ (5)	Female, 35, Chinese	Monomaxillary (Le Fort I)	Cutting the maxillary tuberosity	Asystole	Stopped surgery, atropine
Kim *et al*. (2019) (6)	Male, 23, Asian	Bimaxillary	During fixation of mandibular segments	Arrhythmia followed by bradycardia	Lidocaine IV and glycopyrrolate
Baronos *et al*. (7)	Male, 26, Asian	Bimaxillary (Le Fort I and BSSO)	Use of bite block	Asystole	Removed bite block, glycopyrrolate
Maharaj *et al*. (2020) (8)	Male, 45	Bimaxillary	Down-fracture: During mobilization of the maxilla with the Rowe's disimpaction forceps	Asystole	Stopped surgery, anticipated compressions
Alshalawi *et al*. (2024) (9)​	Male, 32, Saudi	Bimaxillary (Le Fort I and BSSO)	Downfracture of the maxilla	Bradycardia	Atropine
Current case (2024)​	Male, 36, Caucasian	Bimaxillary (Le Fort I and BSSO)	During mandibular nerve stretch (BSSO) // Downfracture: during pterygoid disjunction (Le Fort I)	Bradycardia (BSSO) // Asystole (Le Fort I)	Stopped procedure, atropine (after Le Fort I)

BSSO: Bilateral Sagittal Split Osteotomy.
